# Computed Tomography Pulmonary Angiography (CTPA) Utilization in Suspected Pulmonary Embolism Patients Based on Age-Adjusted D-dimer Thresholds and Pulmonary Embolism Rule-Out Criteria (PERC) Score: A Retrospective Analysis

**DOI:** 10.7759/cureus.79743

**Published:** 2025-02-27

**Authors:** Ahmed Malik, Mohammed Ahmed, Sara Hamid, Eiman Ahmed, Moazzma Ifzaal

**Affiliations:** 1 Acute Internal Medicine, University Hospitals of North Midlands NHS Trust, Royal Stoke University Hospital, Stoke-on-Trent, GBR

**Keywords:** age-adjusted d-dimer, clinical audit, ctpa, nice guidelines, perc score, pulmonary embolism

## Abstract

Computed tomography pulmonary angiography (CTPA) is the gold standard for diagnosing pulmonary embolism (PE), but its overuse can lead to unnecessary radiation exposure and increased healthcare costs. This clinical audit evaluated adherence to National Institute for Health and Care Excellence (NICE) guidelines (NG 158) for CTPA utilization in suspected PE cases. A retrospective analysis of 164 patients at the Royal Stoke University Hospital assessed the application of the pulmonary embolism rule-out criteria (PERC) score in patients aged 18-49 and age-adjusted D-dimer thresholds in those aged 50 and above. Findings revealed that only 14.6% of CTPA scans confirmed PE. Notably, 46.93% of patients under 50 with a PERC score of 0 underwent unnecessary imaging, and 13 patients aged 50 and above with normal age-adjusted D-dimer levels had CTPA, all yielding negative results. These findings indicate a significant overuse of CTPA in low-risk patients. Improving adherence to risk stratification tools through clinician education, decision-support tools, and increased awareness could reduce unnecessary imaging, minimize radiation exposure, and optimize healthcare resource utilization.

## Introduction

Pulmonary embolism (PE) is a potentially life-threatening condition that requires timely diagnosis due to its non-specific presentation [1]. Computed tomography pulmonary angiography (CTPA) is the gold-standard imaging modality [2], but is often overused, leading to unnecessary radiation exposure, increased healthcare costs, and contrast-induced nephropathy [3]. Reducing unnecessary imaging can also decrease patient anxiety and improve patient experience [4]. The National Institute for Health and Care Excellence (NICE) NG 158 guidelines recommend pre-test probability assessment before CTPA to optimize diagnostic pathways [5]. Two key strategies include the PERC score for patients under 50 and age-adjusted D-dimer thresholds for those aged 50 and above [6]. Despite evidence supporting these approaches, adherence remains suboptimal, leading to excessive CTPA use and its potential consequences, such as unnecessary radiation exposure, increased healthcare costs, and contrast-induced nephropathy [4]. Through this clinical audit, we evaluated compliance with NICE guidelines, focusing on appropriately applying the PERC score [7] and age-adjusted D-dimer thresholds. By identifying gaps in adherence, we can significantly enhance diagnostic efficiency, offering a hopeful outlook for the future of PE diagnosis.

## Materials and methods

This retrospective clinical audit was conducted at the Royal Stoke University Hospital between January and February 2024 with a clear goal to assess adherence to NICE guidelines (NG 158) for the use of computed tomography pulmonary angiography in suspected pulmonary embolism cases. A total of 164 adult patients who underwent CTPA during the study period were included. To evaluate guideline adherence, patients were categorized based on age. Those under 50 were assessed for pulmonary embolism rule-out criteria (PERC) score applicability, while patients aged 50 and above were evaluated for adherence to age-adjusted D-dimer thresholds [5].

Patients were rigorously excluded from the audit if they had a Wells score >4 (high pre-test probability), had known cancer or pregnancy, if diagnosed with COVID-19 infection with suspected PE, or had contraindications for CTPA. This stringent approach ensured that the data collected was focused and relevant. Data collection involved extracting demographic, clinical, and imaging details from electronic medical records (EMR) and radiology databases (Table 1). A standardized data extraction form was used to ensure consistency and accuracy; the audit questionnaire items are provided in the Appendices.

**Table 1:** Summary of data variables CTPA: computed tomography pulmonary angiography; PERC: pulmonary embolism rule-out criteria; PE: pulmonary embolism; NICE: National Institute for Health and Care Excellence

Category	Variable
Patient demographics	Age, sex, comorbidities
Clinical presentation	Dyspnea, chest pain, tachycardia, hypoxia
Risk stratification	Wells score, PERC score (<50 years), D-dimer testing (≥50 years)
CTPA outcomes	Positive or negative PE diagnosis
Guideline compliance	Appropriateness of CTPA requests based on NICE recommendations

The audit, a collaborative effort aimed at enhancing patient care, evaluated compliance with NICE guideline NG 158. It specifically assessed the appropriate use of the PERC score in patients under 50 years old, where a score of 0 should rule out the need for further testing, including D-dimer or CTPA [5]. For patients aged 50 and above, adherence to age-adjusted D-dimer thresholds was examined, with CTPA deemed unnecessary if the D-dimer level was below the age-adjusted threshold (age × 10 µg/L) [6]. These measures, a result of our collective work, played a crucial role in determining guideline adherence and optimizing imaging utilization.

## Results

Our audit encompassing 164 patients who underwent CTPA for suspected PE scrutinized their demographic details and clinical presentations. The key aspect of our evaluation was the effective use of risk stratification tools that play a crucial role in adherence to guideline-based diagnostic pathways and patient management optimization. Table 2 summarizes the demographic and clinical characteristics of the 164 patients who underwent CTPA for suspected PE. Most patients (70.1%) were 50 years old or older, while 29.9% were under 50. Gender distribution was relatively balanced, with 51.8% male and 48.2% female patients. These characteristics provide insights into the study population and help evaluate adherence to guideline-based diagnostic pathways [5].

**Table 2:** Demographic and clinical characteristics of participants

Patient characteristics	n (%), total = 164
Age distribution	
<50 years	49 (29.9%)
≥50 years	115 (70.1%)
Gender distribution	
Male	85 (51.8%)
Female	79 (48.2%)

Study findings

The CTPA positivity rate showed that only 14.6% of scans were positive for PE, while the vast majority (85.4%) were negative, highlighting the potential overuse of imaging (Table 3).

**Table 3:** CTPA results for PE CTPA: computed tomography pulmonary angiography; PE: pulmonary embolism

CTPA result	Percentage
Positive for PE	14.6%
Negative for PE	85.4%

It was found that 23 out of 49 patients under 50 years of age (46.93%) had a PERC score of 0 but still underwent CTPA, with all scans returning negative results, indicating a potential overuse of imaging in this group (Table 4) [5].

**Table 4:** Inappropriate CTPA use in patients under 50 years of age (PERC score = 0) CTPA: computed tomography pulmonary angiography; PERC: pulmonary embolism rule-out criteria

Total patients under 50	Patients with PERC score = 0	Percentage	Positive scans	Negative scans
49	23	46.93%	0	23

Table 5 highlights that 13 patients aged 50 and above had a normal age-adjusted D-dimer level but still underwent CTPA. All scans were negative for PE, indicating another area where imaging could have been avoided [6].

**Table 5:** Inappropriate CTPA use in patients aged 50 and above (normal age-adjusted D-dimer levels) CTPA: computed tomography pulmonary angiography

Patients aged 50+	Normal age-adjusted D-dimer levels	Positive scans	Negative scans
13	Yes	0	13

## Discussion

We evaluated adherence to NICE guidelines (NG 158) regarding the appropriate use of CTPA in suspected PE cases [5]. The results highlighted a significant overuse of CTPA, with only 14.6% of scans being positive for PE, indicating a high rate of unnecessary imaging [3]. Two key areas of guideline non-compliance have been identified among patients under 50 with a PERC score of 0; over 50% (23/49) underwent CTPA unnecessarily, with all scans returning negative for PE [6]. Similarly, in patients aged 50 and above with normal age-adjusted D-dimer levels, 13 individuals (11.3%) underwent CTPA despite the low pre-test probability, with no confirmed PE cases. These findings suggest that improved risk stratification and adherence to decision-making tools could significantly reduce unnecessary CTPA requests, thereby minimizing radiation exposure, contrast-related risks, and healthcare costs [4].

The overuse of CTPA is a well-documented issue in clinical practice [3]. Studies have reported CTPA positivity rates as low as 10%-20%, with many negative scans seen in patients with low clinical probability. Similar to our findings, research has shown that failure to apply pre-test probability scores (e.g., PERC, Wells) and age-adjusted D-dimer thresholds contributes significantly to excessive imaging [7]. The PERC rule is particularly relevant for low-risk patients under 50 years of age, as it excludes PE without further testing [6]. Multiple studies have demonstrated that correctly applying the PERC score reduces unnecessary imaging without increasing the risk of missed PE diagnoses [7]. However, this audit found that 52.3% of patients under 50 with a PERC score of 0 still underwent CTPA despite meeting the criteria for rule-out [6].

Age-adjusted D-dimer thresholds are particularly important for patients aged 50 and above [8]. Standard D-dimer thresholds (500 µg/L) have poor specificity in older adults, leading to a high false-positive rate and unnecessary imaging. The age-adjusted threshold (age × 10 µg/L, for patients ≥50 years) improves specificity without compromising sensitivity. Despite this, 13 patients in our study with normal age-adjusted D-dimer levels still underwent CTPA, all with negative results, reinforcing the importance of applying the correct threshold [9].

The findings from this audit highlight opportunities to improve diagnostic pathways for suspected PE by enhancing adherence to risk stratification tools. Ensuring the systematic application of the PERC score in patients under 50 years and promoting the routine use of age-adjusted D-dimer thresholds in older patients are key measures that could reduce unnecessary imaging [6,10]. Implementing decision-support systems in EMR could prompt clinicians when CTPA is requested without appropriate pre-test assessment [11]. Additionally, education and training sessions for junior doctors and Acute Medicine clinicians on NICE guideline adherence would reinforce best practices [12].

Minimizing unnecessary CTPA requests has important clinical and economic implications. Each CTPA exposes patients to approximately 10-15 mSv of radiation, equivalent to several years of natural background exposure [4]. Avoiding unnecessary imaging reduces patient risk and optimizes resource allocation, improving overall efficiency in emergency and radiology departments [5].

Limitations

Despite its valuable insights, this audit has some limitations. As a single-centre study, findings may not be generalizable to other hospitals with different patient populations and referral patterns [3]. The retrospective design also relied on medical records, which may have missing or incomplete documentation of clinical decision-making [12]. The sample size (n=164) was relatively small; more extensive studies with prospective validation would provide more robust conclusions [9].

Future directions and recommendations

We recommend several quality improvement measures to improve adherence to NICE guidelines (NG 158) and optimize CTPA utilization. Implementing a clinical decision-support tool that integrates automated prompts to flag patients with a PERC score of 0 or normal age-adjusted D-dimer levels before approving CTPA requests could enhance guideline adherence [11]. Regular training sessions for Acute Medicine staff on appropriate PE risk stratification and imaging indications would further support best practices [12]. Finally, conducting a follow-up audit after implementing these interventions would allow for prospective monitoring and assessment of improvements in guideline adherence and reductions in unnecessary CTPA use.

## Conclusions

This clinical audit highlights the significant overuse of CTPA in suspected PE cases, with a high rate of negative scans, particularly among low-risk patients who met the established rule-out criteria. Non-adherence to the PERC score in patients under 50 and failure to apply age-adjusted D-dimer thresholds in those aged 50 and above contributed to unnecessary imaging, increasing radiation exposure, healthcare costs, and resource burden. Strengthening adherence to NICE guidelines through mandatory documentation, decision-support tools, clinician education, and regular audits is essential to optimize imaging practices, enhance patient safety, and improve healthcare efficiency.

## Appendices

**Table 6:** Clinical audit questionnaire CTPA: computed tomography pulmonary angiography; PERC: pulmonary embolism rule-out criteria; NICE: National Institute for Health and Care Excellence; SOB: shortness of breath; CXR: chest X-ray

Section	Questionnaire items
1. Patient demographics	a. Age
	b. Gender
	c. Past medical history (e.g., cardiovascular disease, pulmonary disease)
	d. Risk factors for venous thromboembolism (VTE) (e.g., immobility, recent surgery)
2. Clinical presentation	a. Chief complaint (chest pain, SOB, both)
	b. Onset and duration of symptoms
	c. Associated symptoms (e.g., hemoptysis, leg swelling)
	d. Initial clinical assessment (e.g., vital signs, physical examination findings)
3. Pre-CTPA investigations and management	a. Documentation of clinical suspicion for pulmonary embolism (PE)
	b. Use of risk stratification tools (Wells' score and PERC rule)
	c. Performance of D-dimer test if indicated
	d. Troponin result
	e. ECG findings
	f. CXR findings
	g. Any initial management strategies before CTPA (e.g., oxygen therapy, anticoagulation)
	b. Use of risk stratification tools (Wells' score and PERC rule)
4. Alternative diagnosis considered	Documentation of differential diagnoses
5. CTPA utilization	a. Indication for CTPA
	b. Documentation of contraindications or precautions prior to CTPA
	c. Radiological findings on CTPA report (positive, negative, indeterminate)
6. Post-CTPA management	a. Interpretation and action taken based on CTPA findings
	b. Initiation of anticoagulation therapy if PE diagnosed
	c. Referral to appropriate specialty services (e.g., cardiology, pulmonology)
	d. Documentation of patient disposition (admission, discharge)
7. Documentation and follow-up	a. Accuracy and completeness of CTPA report documentation
	b. Communication of CTPA results to the patient
	c. Follow-up plan for patients with negative or indeterminate CTPA results
	d. Documentation of any adverse events related to CTPA or subsequent management
8. Compliance with NICE guidelines	a. Adherence to NICE guidelines for CTPA utilization in patients with chest pain or SOB
	b. Identification of any deviations from NICE recommendations and reasons for such deviations
